# BMRMI Reduces Depressive Rumination Possibly through Improving Abnormal FC of Dorsal ACC

**DOI:** 10.1155/2022/8068988

**Published:** 2022-04-04

**Authors:** Ming-Hao Yang, Zhi-Peng Guo, Xue-Yu Lv, Zhu-Qing Zhang, Wei-Dong Wang, Jian Wang, Lan Hong, Ying-Na Lin, Chun-Hong Liu

**Affiliations:** ^1^Beijing Hospital of Traditional Chinese Medicine, Capital Medical University, Beijing, China; ^2^Department of Psychology, Guang'anmen Hospital, China Academy of Chinese Medical Sciences, Beijing, China; ^3^Beijing Institute of Chinese Medicine, Beijing, China

## Abstract

Rumination is a common symptom of major depressive disorder (MDD) and has been characterized as a vulnerability factor for the onset or recurrence of MDD. However, the neurobiological mechanisms underlying rumination and appropriate treatment strategies remain unclear. In the current study, we used resting-state functional magnetic resonance imaging to investigate the effects of body-mind relaxation meditation induction (BMRMI) intervention in MDD with rumination. To this aim, we have recruited 25 MDD and 24 healthy controls (HCs). Changes in functional connectivity (FC) of the anterior cingulate cortex (ACC) subregion and the scores of clinical measurements were examined using correlation analysis. At baseline, MDD showed stronger FC between the right dorsal ACC (dACC) and right superior frontal gyrus than did the HC group. Compared to baseline, the HC group showed a significantly enhanced FC between the right dACC and right superior frontal gyrus, and the MDD group demonstrated a significantly weaker FC between the left dACC and right middle frontal gyrus (MFG) after the intervention. Furthermore, the FC between the right dACC and right superior frontal gyrus was positively associated with rumination scores across all participants at baseline. The above results indicate that BMRMI may regulate self-referential processing and cognitive function through modulating FC of the dACC in MDD with rumination.

## 1. Introduction

Major depressive disorder (MDD) is a severe mental disease, which is characterized by anhedonia, repetitive rumination, and cognitive impairment [1]. The lifetime prevalence rate of MDD is about 11–15%, and it affects approximately 5–6% of people worldwide each year [2–4]. One risk factor for MDD onset or recurrence is depressive rumination, which is conceptualized as passive and repetitive attention to one's negative aspects and a tendency to focus on their possible causes and negative consequences [5, 6]. Moreover, there is evidence that higher levels of rumination are correlated with other clinical outcomes, such as a slower treatment response [7, 8] and inferior initial remission [9, 10]. Recent studies have reported that body-mind relaxation meditation induction (BMRMI) can significantly reduce depressive rumination [11]. However, the mechanisms underlying it is still not clear.

BMRMI, a kind of mindfulness meditation, has been found to reduce anxious and depressive symptoms, without any side effects [12]. BMRMI resembles yoga; in that, it promotes the ability to change physiological behavior during a guided relaxation process and also facilitates positive emotional experiences [13]. More importantly, BMRMI involves listening to musical melody and relaxation instructions, which enable individuals to balance their physical and mental state and promote the recovery of cognitive function and negative emotions [11]. Clinical reports have indicated that mindfulness meditation is beneficial for developing alternative responses to negative thoughts and reducing habitual rumination [14]. Neuroimaging studies have shown that BMRMI can affect brain regions which were related with attention and emotional processing, such as the anterior cingulate cortex (ACC), frontal cortex, insula, and sensorimotor cortex [15].

In recent years, neuroimaging studies have found that increased rumination was related to altered activation and connectivity of the default mode network (DMN) in MDD [16–19]. The DMN comprises the precuneus, medial prefrontal cortex (mPFC), and posterior cingulate cortex (PCC) and is implicated in self-referential processing, emotion regulating, and cognitive improving [20–22]. Cooney et al. (2010) found that there is significantly more activation in mPFC and PCC in patients with MDD compared to healthy controls (HCs) during rumination induction [17]. Recent studies have also indicated that depressive rumination is correlated with activity in a range of regions (e.g., the ACC, amygdala, and hippocampus), which are known to be implicated in attention control and autobiographical memory [23, 24]. Kühn et al. (2012) demonstrated that ruminative thoughts were negatively associated with gray matter density and activity in the inferior frontal gyrus and ACC [25]. Using independent component analysis, rumination scores were found to be associated with increased functional connectivity (FC) between the ventral mPFC and ventral ACC [21]. Moreover, ACC metabolic activity and connectivity can predict the response to antidepressants and other therapies [26–28]. The ACC therefore seems to play a particularly major role in rumination, treatment response, and the remission of MDD.

Based on its functional heterogeneity and cytoarchitecture, Margulies et al. (2007) divided the ACC into 16 seed regions that are distributed in two parallel rows [29]. Kelly et al. (2009) proposed that the ACC can be subdivided into five seeds in each hemisphere, each of which is associated with five respective functions [30]. The subregions of the ACC include the caudal ACC (cACC), dorsal ACC (dACC), rostral ACC, perigenual ACC (pgACC), and subgenual ACC [29]. The cACC is commonly thought to function in tandem with fronto-parietal regions and has been proposed to integrate sensorimotor processes [31]. The dACC activation has been associated with autobiographical memory and cognitive control and is proposed to act “circuit hub” in top-down pathway [32, 33]. The rACC exhibits patterns of activity that are correlated with the amygdala, hippocampus, ventromedial PFC, and posterior cingulate cortex, which have been implicated in affective processing [34, 35]. The pgACC has been demonstrated to consist a component of emotion regulation network and implicated in modulating the increased inner attention to ruminative thinking of patients with MDD [36, 37]. The sgACC is involved in autonomic control and self-referential processing via connection with the anterior part of DMN [38]. In this study, we used these ACC subregions as the seed regions for resting-state functional magnetic resonance imaging (rs-fMRI) to investigate the neural mechanism underlying the effect of a short-term BMRMI intervention in MDD with rumination. We hypothesized that BMRMI treatment would strengthen FC in the ACC subregions in MDD with rumination. We also expected that FC changes would be correlated with the clinical variables.

## 2. Methods

### 2.1. Participants

Participants were 25 patients with MDD who had been diagnosed by administration of the DSM-IV by two qualified psychiatrists [39], and 24 HCs were also recruited by advertisement from the local community. Education levels and years of education were determined using self-reported information from participants [40]. All recruited patients with MDD met the following criteria: Hamilton Depression Rating Scale (HAMD) score ≥ 17; no stable drug treatment; no other psychiatric symptoms or acute physical disease; and no history of other interventions, especially for mindfulness meditation, qigong practice, or yoga. The exclusion criteria for HCs are as follows: a history of mindfulness meditation in the last 2 months, head injury, no history of alcohol or drug abuse, pregnancy, and a family history of psychiatric illnesses. This study was approved by the Medical Ethics Committee of Guang'an Men Hospital, China Academy of Chinese Medical Sciences (Beijing, China; ethical approval number 2017-056-KY-01), and informed consent was signed from all participants before study enrollment.

### 2.2. Measures

The severity of depressive and anxious symptoms was assessed in all participants by two psychiatrists using the HAMD and Hamilton Anxiety Rating Scale (HAMA). The level of rumination was assessed in all participants using the Automatic Thoughts Questionnaire (ATQ) and Ruminative Responses Scale (RRS) clinical measurements. The ATQ is a commonly used to evaluate the frequency of automatic occurrence of negative self-thoughts [41]. The RRS evaluates repetitive responses to depressed emotions and passive self-thoughts that focused on a person's negative feelings, relative symptoms, or their causes and consequences [42]. These measures were not implemented after the BMRMI intervention.

### 2.3. Intervention

BMRMI is composed of harmonious background music and relaxation-inducing passages. The background music is called “Saishangqu,” which is soft, slow Chinese classical music played by zither [11]. The relaxation guide passage was conducted in Mandarin by a female speaker, comprising two parts: (1) phrases that induce whole-body relaxation, such as “relax your muscles from top to bottom,” and (2) phrases that induce mind relaxation, such as “feel your body relax and take some downtime.” The BMRMI treatment session lasted 15 minutes. All participants were scanned at both baseline and immediately after the BMRMI intervention.

### 2.4. rs-fMRI Data Acquisition

All participants underwent brain scans on a 1.5 T GE Signal scanner (GE Healthcare, Piscataway, NJ, USA) in Guang'an Men Hospital. The functional images were acquired in 41 axial slices from the echo planar imaging sequence (slice thickness = 3 mm, gap = 0.5 mm, repetition time (TR) = 2500 ms, echo time (TE) = 30 ms, matrix size = 64 × 64 × 20, flip angle = 90°, and field of view = 24 × 24 cm). All participants underwent a 360-second resting-state scan, and data were acquired 144 time points. All participants were instructed to keep their eyes closed in quiet state while scanning, and foam pads were used to immobilize the head. After scanning, each subject was recorded whether they had not fallen asleep or had been distracted by something during the scan.

### 2.5. Image Data Preprocessing

The image data were preprocessed using the Data Processing Assistant for Resting-State fMRI Advanced Edition (DARSF 4.0) based on MATLAB 2014a software. The first 10 volumes from both scanning sessions were removed. The remaining volumes for each subject were processed to correct head motion and calculate slice timing, normalized to the standard Montreal Neurological Institute (MNI) template, and smoothed using a 6 × 6 × 6 full-width at half-maximum Gaussian kernel. Data were bandpass-filtered at 0.01–0.08 Hz and linearly detrended. The nuisance signals from white matter, cerebrospinal fluid, and six parameters of head motion were also regressed out. Any participants who had a translation larger than 2 mm and greater than 2° in any angular dimension were discarded from subsequent analysis [29, 30]. Two subjects in the MDD group were discarded due to excessive head motion > 2.0 mm during the MRI scanning. The mean framewise displacement was not significantly different between baseline (0.124 ± 0.085) and after BMRMI treatment (0.133 ± 0.014) in the MDD group (*T* = −0.488, *p* = 0.63).

### 2.6. Seed Region of Interest Functional Analysis

**Figure 1:**
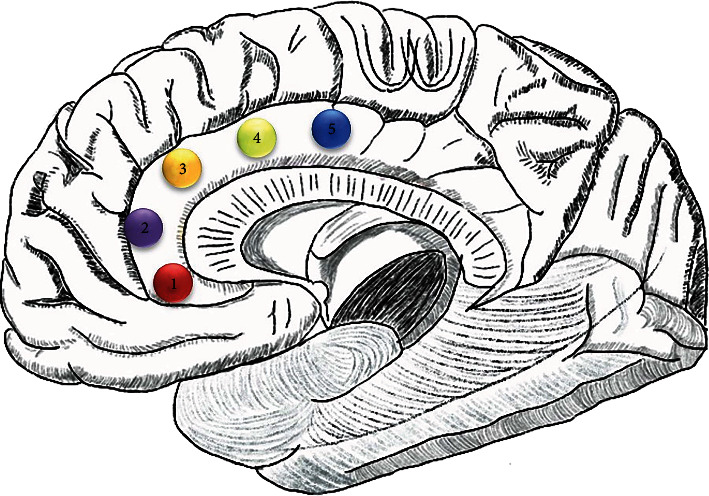
The locations of the ACC subregions in each hemisphere. 1: cACC; 2: dACC; 3: rACC; 4: pgACC; 5: sgACC. Abbreviations: ACC: anterior cingulate cortex; cACC: caudal ACC; rACC: rostral ACC; dACC: dorsal ACC; pgACC: pregenual ACC; sgACC: subgenual ACC.

The ACC was divided into five subregions for each hemisphere (Figure 1). The ACC subregions included the cACC (MNI = ±5, −10, 37), dACC (MNI = ±5, 10, 33), rostral ACC (MNI = ±5, 27, 21), pgACC (MNI = ±5, 47, 11), and sgACC (MNI = ±5, 34, −4) [29, 30]. These seeds were selected as spheres with a 6 mm diameter in the peak center of brain regions, symmetrically in both hemispheres [43]. For the ACC subregions in both hemispheres, the average time series of each region of interest were extracted. The *p*-correlation coefficients were computed between each ROI mean time coursed and that of each voxel of the whole brain. Fisher's *r*-to-*z* transformation was used to convert FC results to *z*-values to improve the normality.

### 2.7. Statistical Analyses

All statistical analyses of the demographic data and clinical measurements were performed using SPSS version 25.0 (SPSS, Inc., Chicago, IL, USA). Baseline differences in demographic data and clinical measurements between the MDD group and HCs group were analyzed using a two-sample *t*-test, and sex differences were analyzed using the *χ*^2^ test. After verifying the normality and homogeneity of the variance of MRI data, a repeated-measures ANOVA was applied to examine the FC of ACC subregion differences between two groups in both before and after treatment, with age, education, and sex as covariates [44]. After using Gaussian random field theory to correct the results of statistically significant FC mapping, the voxel-level threshold was *p* < 0.001, and the joint cluster-level threshold was *p* < 0.05. Next, the correlation between the mean change values of FC in ACC subregions and scores of clinical scales was assessed in both groups and in all participants before BMRMI.

## 3. Results

### 3.1. Demographic Data and Clinical Measurements

The are no significant differences in age (*t* = 0.10, *p* = 0.91), education (*t* = −1.22, *p* = 0.23), or sex (*χ*^2^ = 0.07, *p* = 0.792) between two group. Significant between-group differences were found in the HAMD, HAMA, ATQ, and RRS scores (for details, see Table 1).

### 3.2. ANOVA Differences in ACC Subregion Seed-Based FC

**Figure 2:**
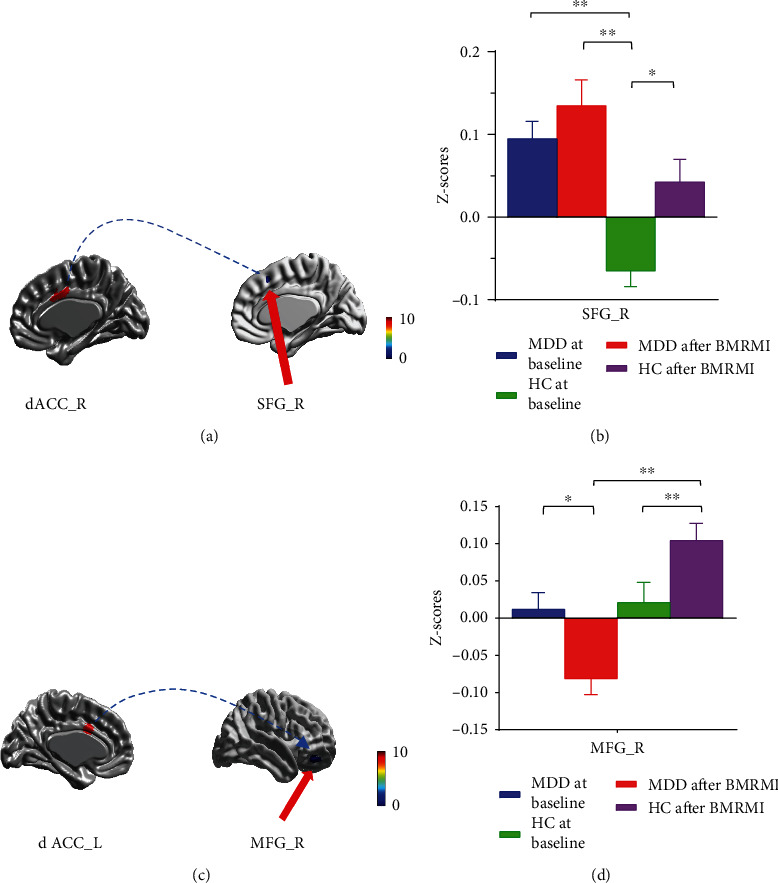
Aberrant functional connectivity of ACC subregions in MDD group and HCs group at baseline and after BMRMI. (a, b) The MDD group demonstrated increased functional connectivity between the right dACC and right SFG compared to the HC group at baseline (*p* < 0.01). The HC group demonstrated increased functional connectivity between the right dACC and right SFG after BMRMI treatment (*p* < 0.05). (c, d) The MDD group demonstrated decreased functional connectivity between the left dACC and right MFG after BMRMI treatment (*p* < 0.05). The HC group showed enhanced functional connectivity between the left dACC and right MFG after BMRMI treatment (*p* < 0.01). BMRMI: body-mind relaxation meditation induction; dACC: dorsal anterior cingulate; SFG: superior frontal gyrus; MFG: middle frontal gyrus; MDD: major depressive disorder; HC: healthy control; HAMD: Hamilton Depression Rating Scale; ATQ: Automatic Thoughts Questionnaire; RRS: Ruminative Responses Scale. ^∗^*p* < 0.05; ^∗∗^*p* < 0.01.

The region of interest analysis results revealed aberrant FC between MDD group and HC group at baseline and after intervention in the right dACC and right SFG and left dACC and right middle frontal gyrus (MFG) (Table 2). There was a significant difference in FC between the right dACC and right SFG in the MDD group relative to the HC group before BMRMI. After BMRMI treatment, we found a stronger FC between the right dACC and right SFG in both groups relative to baseline. We also found that FC between the dACC and MFG decreased significantly after the intervention within the MDD group; conversely, the FC increased significantly after the intervention within the HC group (Figure 2).

### 3.3. Correlation Analysis

**Figure 3:**
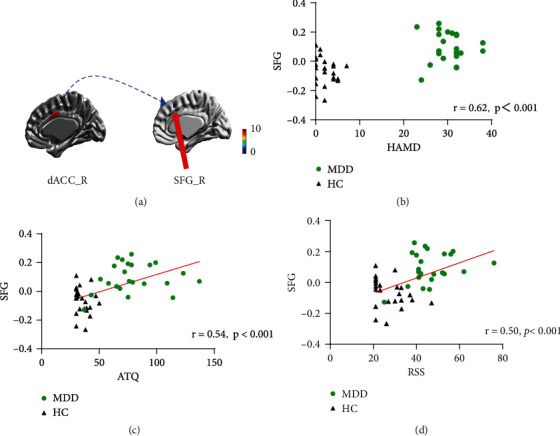
The correlation between the aberrant seed-based functional connectivity and clinical measurements at baseline. (a) Brain regions showed an aberrant functional connectivity of the right dACC with right SFG between MDD group and HCs group at baseline. (b–d) The voxel-wise correlation analyses revealed that a stronger FC between the right dACC and right SFG was correlated with higher HAMD, ATQ, and RRS scores in all participants at baseline. dACC: dorsal anterior cingulate; SFG: superior frontal gyrus; MDD: major depressive disorder; HC: healthy control; HAMD: Hamilton Depression Rating Scale; ATQ: Automatic Thoughts Questionnaire; RRS: Ruminative Responses Scale.

The correlation between the subregions of ACC FC and clinical measurements in all participants at baseline are shown in Figure 3. Correlation analysis revealed that the increased FC between the right dACC and right SFG was significantly correlated with the higher scores of RRS (*r* = 0.50, *p* < 0.01), ATQ (*r* = 0.54, *p* < 0.001), and HAMD (*r* = 0.62, *p* < 0.001) in all participants before BMRMI.

## 4. Discussion

The present study used rs-fMRI to investigate the effect of BMRMI intervention on FC changes in ACC subregions in patients with MDD. At baseline, the MDD group showed stronger FC between the right dACC and right SFG than did the HC group. After BMRMI treatment, we found an enhanced FC between the right dACC and right SFG in both groups compared to baseline, but this difference was not significant within the MDD group. However, we also found a different connectivity pattern between the left dACC and right MFG in both the MDD group and HC group after BMRMI treatment. In particular, the strength of FC between the right dACC and right SFG was positively correlated with HAMD, RRS, and ATQ scores across all participants before BMRMI. These findings support the notion that BMRMI increases FC between the dACC and SFG through modulating attentional control, and these changes may play a considerable role in MDD with rumination.

In comparison with HC group, MDD patients showed a stronger FC between the right dACC and right SFG at baseline. Moreover, the increased FC between the right dACC and right SFG was associated with higher rumination scores across all participants at baseline. Previous studies have suggested the differential activity or abnormal FC of ACC subregion activity in MDD is substantively different to that of healthy individuals [45]. Numerous research has demonstrated that the ACC has high functional coupling with core part of DMN in MDD with rumination [46–48]. For example, the decreased FC between the sgACC and right MFG has been found to be associated with higher RRS scores [49, 50]. Negative self-focused thought has also been reported to be positively correlated with pgACC connectivity with the dorsolateral PFC [51]. Another study detected that FC between the dACC and precuneus was positively correlated with rumination in patients with MDD [52]. Connectivity changes between the dACC and dorsal mPFC have also been observed in the remission of patients with MDD who are prone to rumination [18]. Importantly, the dACC is not only a hub in a network of regions involving cognitive functioning but forms a part of the salience network, which has been demanded in the processes of attentional control and monitoring conflict [52–54]. Specifically, the correlation between the increased FC of the dACC and SFG with RRS scores found in the present study indicates that depressive rumination is associated with attentional control impairments. Combined with the above results, our findings expand our understanding of abnormal FC between ACC subregions and highlight the role of the DMN as a neural mechanism underlying rumination.

After BMRMI treatment, FC between the right dACC and right SFG increased significantly in the HC group compared to baseline; however, no such significant increase in FC was found in the MDD group. Given that our intervention was a single brief session of BMRMI and clinical measurement scales were used to evaluate outcomes, we missed the correlation between the difference FC and the change of clinical measurements after treatment, so we need to interpret the results with caution. Our findings may indicate that patients with MDD require greater effort or more adequate treatment to improve higher cognitive processes and avoid negative emotion. Indeed, previous studies have also demonstrated that mindfulness-based intervention has a beneficial effect on cognitive function. For instance, 1 month of music intervention has been found to strengthen FC between the right middle temporal gyrus [44] and between the dorsal anterior insula and ACC [55] in patients with schizophrenia. After an 8-week mindfulness-based intervention in healthy individuals, Hölzel et al. (2010) found an increased density of gray matter in the PCC and temporo-parietal junction [56]. After an 11-hour body-mind training meditation in patients with schizophrenia, Tang et al. (2018) found increased white matter tract integrity connecting the vACC and dACC [57]. Using different musical and nonmusical emotion-stimuli, Lepping et al. (2016) found that patient with MDD showed the strongest activation by negative nonmusical stimuli and less activation for positive musical in the dACC after each stimulus, while the HC group exhibited greater dACC activation in response to all music than to all nonmusical stimuli [58]. Regardless of whether an intervention is long-term or short-term, previous studies have reported that mindfulness-based therapy can improve cognitive functioning. However, we did not find a change in dACC and SFG FC after BMRMI in the MDD group, most likely because our intervention comprised a single brief session of BMRMI. In the future, we will implement a complete treatment routine and collect the relevant clinical measurements to further investigate the effect of BMRMI intervention and expand our understanding in mechanism of MDD.

We also found decreased FC between the left dACC and right MFG in the MDD group after BMRMI compared to baseline. The MFG is a major part of dorsolateral PFC, which is a component of the executive control network and involved in extensive attentional regulation, decision-making, and semantic processing [59]. Lepping and colleagues have reported that patients with MDD showed a smaller response to positive music in the dACC [58]. Our results extend this work and indicate that patients with MDD show a blunt response to positive emotional stimuli and require more attention and/or executive control to control competing negative information.

### 4.1. Strengths

First, the main objective of this study was not only to determine differences in FC between patients with MDD and HCs but also to identify the specificity of these aberrant FC patterns of ACC subregions observed in neuroimaging studies. Second, our results further confirmed the relationship between depressive rumination and attentional control impairments. Third, this study was defined as a preliminary study to investigate the possible effects of BMRMI in MDD with rumination, which could provide some basic data for assessing the long-term intervention in future studies.

### 4.2. Limitations

The present study has several limitations. First of all, the sample size is relatively small; we will expand the sample size to improve the reliability of data. Second, the total scanning duration was only 144 time points; in the future, we will use a longer sequence to improve measurement reliability (Birn et al., 2013). Third, only one routine therapy session was implemented in our study. Fourth, we did not measure rumination or depression severity after BMRMI treatment, so we could not ascertain whether FC changes in ACC subregions were associated with the changes of symptoms in MDD. Future studies should implement a complete treatment routine and collect the relevant clinical measurements after treatment. It would be meaningful to conduct a longitudinal study to investigate whether the altered FC in brain networks can predict the clinical outcome of BMRMI.

## 5. Conclusions

Our findings demonstrate that an altered FC between the dACC and SFG is involved in rumination pathophysiology in patients with MDD. Furthermore, we found that BMRMI could positively improve the functional hub of the dACC through modulating attentional control in patients with MDD with rumination. Our results may provide an objective evidence supporting the effect of BMRMI intervention in regulating self-referential processing and in cognitive functioning.

## Figures and Tables

**Table 1:** Group demographics and clinical measurements.

Measure (mean ± SD)	MDD (*n* = 23)	HC (*n* = 24)	Statistic value	*p* value
Sex (male/female)	5/18	6/18	0.07	0.792*^Δ^*
Age	35.43 ± 9.28	35.17 ± 7.83	0.1	0.915^∗^
Education	13.65 ± 3.08	15.08 ± 4.78	-1.22	0.23^∗^
HAMD	30.30 ± 3.63	2.13 ± 2.05	32.54	<0.0001^∗^
HAMA	17.39 ± 6.39	1.21 ± 1.31	12.13	<0.0001^∗^
ATQ	79.09 ± 24.63	35.42 ± 6.00	8.43	<0.0001^∗^
RRS	46.39 ± 10.27	27.29 ± 7.09	7.44	<0.0001^∗^

Abbreviations: MDD: major depressive disorder; HC: healthy control; HAMD: Hamilton Depression Rating Scale; HAMA: Hamilton Anxiety Rating Scale; ATQ: Automatic Thoughts Questionnaire; RRS: Ruminative Responses Scale. ^∗^*p* values for two-sample *t*-tests, and *^Δ^p* values for chi-square test.

**Table 2:** Between-group seed-based FC differences in the ACC subregions before and after BMRMI.

ROI regions	Hemisphere	Brain regions	Side	Brodmann areas	Coordinates			Voxels	Peak intensity
					*X*	*Y*	*Z*		
dACC	R	Superior frontal gyrus	R	32	21	39	51	13	10.71
dACC	L	Middle frontal gyrus	R	11	30	42	-18	19	8.81

Abbreviations: ANOVA: analysis of variance; ACC: anterior cingulate cortex; FC: functional connectivity; MDD: major depressive disorder; HC: healthy control; BMRMI: body-mind relaxation meditation induction; ROI: regions of interest; dACC: dorsal anterior cingulate cortex; R: right; L: left.

## Data Availability

Sorry, we cannot provide relevant data because it involves the privacy of patients.
